# Stereopsis without correspondence

**DOI:** 10.1098/rstb.2021.0449

**Published:** 2023-01-30

**Authors:** Jenny C. A. Read

**Affiliations:** Biosciences Institute, Newcastle University, Newcastle upon Tyne, Tyne and Wear UNE2 4HH, UK

**Keywords:** stereopsis, evolution, binocular vision, computational neuroscience

## Abstract

Stereopsis has traditionally been considered a complex visual ability, restricted to large-brained animals. The discovery in the 1980s that insects, too, have stereopsis, therefore, challenged theories of stereopsis. How can such simple brains see in three dimensions? A likely answer is that insect stereopsis has evolved to produce simple behaviour, such as orienting towards the closer of two objects or triggering a strike when prey comes within range. Scientific thinking about stereopsis has been unduly anthropomorphic, for example assuming that stereopsis must require binocular fusion or a solution of the stereo correspondence problem. In fact, useful behaviour can be produced with very basic stereoscopic algorithms which make no attempt to achieve fusion or correspondence, or to produce even a coarse map of depth across the visual field. This may explain why some aspects of insect stereopsis seem poorly designed from an engineering point of view: for example, paying no attention to whether interocular contrast or velocities match. Such algorithms demonstrably work well enough in practice for their species, and may prove useful in particular autonomous applications.

This article is part of a discussion meeting issue ‘New approaches to 3D vision’.

## 1. Introduction

Gaining information about the 3D structure of the world from two-dimensional retinal images is a major challenge for any visual system. It has long been appreciated that our binocular vision aids with this task [1–4], but it was generally assumed that this occurred through sensing one's vergence: the rotation made by the two eyes so that both are fixating a given object. It was not until 1838, when Wheatstone published a description of his stereoscope in a previous volume of this publication [5], that scientists realized depth perception can be enhanced by comparing the slightly different views of the same object in the two eyes, independent of any additional information arising from knowledge of eye posture. This ability is known as stereoscopic vision or stereopsis [5].

Perhaps because it was recognized so late, our thinking about stereoscopic vision seems to have been more than usually anthropocentric: ‘heavily influenced by the complex neuronal principles realized in higher vertebrates’ [6]. A common early view was that stereopsis was indeed ‘confined solely to man and to a few of the higher animals in whom the eyes are placed side by side’ [7]. This assumed link between stereopsis, front-facing eyes and a predatory lifestyle has been persistent and influential, though there is little evidence to support it [8]. Front-facing eyes do indeed seem to be associated with nocturnal visual predators [9,10], but stereopsis is far more widespread, while on theoretical grounds, lateral eyes are arguably better for stereopsis, since a wide baseline improves range and precision. A more principled early assumption was that stereopsis must be restricted to mammals with a partial decussation of the optic nerve, since information from both eyes is then available to each cortical hemisphere [11]. On this basis, the ophthalmologist Edward Treacher Collins inferred stereopsis in the horse long before that was demonstrated empirically [12], suggesting that ‘binocular stereoscopic vision … must be of the greatest assistance to it in the estimation of size and distance in jumping’ [13]. Many of the selective advantages suggested for stereopsis in humans would be useful to other species: manual dexterity and tool use [11,13], arboreal locomotion [13,14], bipedal locomotion [15], accurate perception of the ground plane [15–17] and/or visual predation [9,10,13], whether by enabling the precise judgement of distances and/or by breaking camouflage [18]. Indeed, we now know that as well as existing in lateral-eyed mammals such as horses, sheep and mice [12,19–22], stereopsis is also found in many animals with a full initial decussation, such as barn owls, falcons and toads [23–25]. Most recently, it has now also been demonstrated in two widely different invertebrate taxa: insects and cephalopods [26–28]. Thus, stereopsis appears to be extremely widespread in the animal kingdom. Its distribution indicates that it must have evolved independently multiple times, suggesting that it is valuable in a wide range of ecological niches [8,29,30].

Biological forms of depth perception contrast strikingly with current machine approaches. In current mobile autonomous devices, depth perception is most often achieved by teleceptive active sensing, where energy generated by the device is transmitted into the environment and then received back without contact, for example using structured light, LIDAR, radar or ultrasound. However, in the animal kingdom, passive sensing using ambient light is far more common. This is clearly not because organic systems are incapable of evolving teleceptive active sensing. Active sensing using ultrasound or electrolocation has evolved in several taxa [31,32], but only where vision is not usable due to turbid water (e.g. marine mammals, fish) or lack of light (e.g. bats). This suggests that in biological systems, adequate distance information can be provided by passive vision, avoiding the higher energy costs of active sensing. Clearly, it would be highly beneficial to mobile autonomous devices, especially those where power consumption is a key constraint, to be able to replicate the depth sensing performance of biological visual systems, and this must include stereopsis.

Most current machine stereo algorithms are modelled more or less explicitly on human stereo vision. Even the benchmarks developed to evaluate them, such as the accuracy of a depth map extracted from a single pair of stereo-images [33], are inspired by human vision. Praying mantis stereopsis, for example, detects the disparity between regions of the scene where things are changing [34,35], and thus would fail such a benchmark completely. Human stereopsis gives detailed and highly precise information about relative depth in the central 15° or so of our visual field; it can reveal objects which are perfectly camouflaged in the monocular image by detecting depth discontinuities at their edges [18]; and it can support complex percepts such as multiple layers of transparency [36]. This highly sophisticated form of stereopsis is challenging and computationally expensive to achieve.

Given the recent increase in knowledge of non-primate stereopsis, it is timely to seek inspiration from forms of stereopsis very different from our own. In this paper, I want to use recent knowledge about insect stereopsis to challenge the idea that stereopsis requires sophisticated algorithms which derive a projective transformation between image-points in left and right eyes and use this to deduce a depth map across the visual field. Evolutionarily, all that is required is useful behaviour. So, there may be more efficient mappings from retinal images to useful behaviour, which do not require the computation of disparity or depth. As discussed in the introduction to this volume, there is a long tradition of such an ‘active vision’ approach, aiming for a ‘direct coupling of perception and action, without an explicit 3D intermediary’ [37]. But in the context of stereopsis, ‘without an explicit 3D intermediary’ has tended to mean making inferences about scene properties (e.g. surface slant) directly from disparity [38–40]. Little attention has been paid to an even more basic approach, where even disparity is not explicitly encoded.

In this paper, I will begin with a fairly detailed review of the conventional approach, in which disparity is encoded in the activity of a population of disparity sensors, each tuned to a particular disparity and visual direction. I will discuss the challenges of achieving stereo correspondence and binocular fusion, distinguishing between disparity sensors which achieve ideal, strong, weak or no correspondence. I will consider how local mechanisms have to interact across wider regions of the visual field in order to achieve reliable correspondence, and briefly review how this problem is approached in machine stereo algorithms and what we know about how it happens in the human brain. In the second part of this paper, I will contrast this with two different forms of stereopsis inspired by insect vision. One is conceptually similar to previous proposals [6,41–43] regarding praying mantis predatory strikes, which was tested for the first time in our recent paper [44]. The other was proposed recently as a simple way to include stereo information when selecting a target [8], and is tested for the first time in this paper. Neither of these algorithms achieves stereo correspondence or binocular fusion of multiple objects, or even a computation of binocular disparity. The algorithms do *exploit* disparity, but they do not *estimate* disparity unconfounded with other stimulus dimensions. Nevertheless, simulation results suggest that they suffice to produce useful stereoscopic behaviour, such as preferentially orienting towards the nearer of two competing targets. Both algorithms are inspired by the praying mantis and replicate key aspects of its behaviour, justifying the claim that the behaviour is indeed useful. Both algorithms would need further modifications to fully account for mantis behaviour. The aim here is not so much to provide a complete account of praying mantis vision, as to demonstrate that these very basic, non-correspondence stereo algorithms suffice for useful behaviour.

## 2. Stereopsis and correspondence

**Figure 1.**
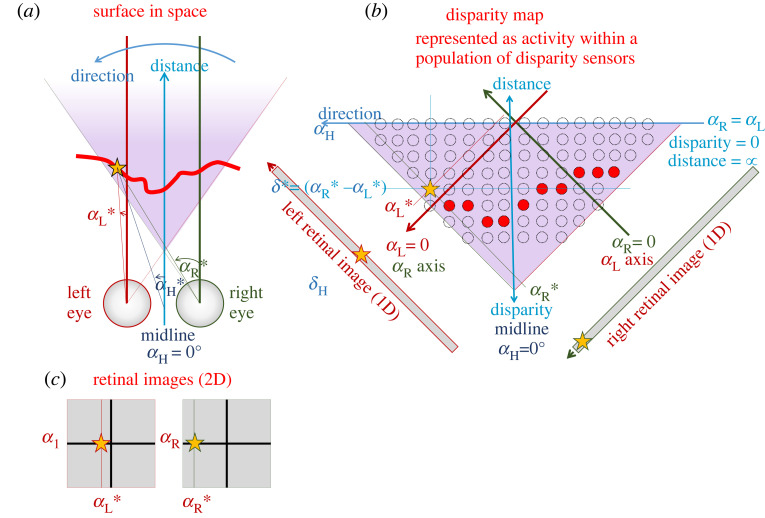
Geometry of stereopsis in headcentric coordinates, appropriate for fixed eyes. (*a*) A horizontal cross-section through the eyes and the space in front of the animal, at zero elevation. The purple shaded region shows the binocular overlap, i.e. the region of space visible to both eyes. This is triangular if the field of view for each eye extends further temporally than nasally. The orange star marks an example point on an example surface. The azimuthal angles *α*_R_, *α*_L_ indicate how far each location is from the direction ‘straight ahead’ in each eye. We define the headcentric azimuth to be the average of these: *α*_H_ = (*α*_R_ + *α*_L_)/2, while the disparity is their difference: *α*_R_ − *α*_L_. The angles marked with * show the value of these angles for the point marked with the star. (*b*) The same space replotted in terms of the angular location, so that the Cartesian axes are the azimuth in each eye. The red, green arrows mark the axes of *α*_L_, *α*_R_, respectively. The vertical axis is headcentric disparity *δ* = *α*_R_ − *α*_L_, and the horizontal axis is proportional to headcentric azimuth or visual direction. This runs right to left as a consequence of our coordinate system: we use a right-handed coordinate system in which the *Z*-axis points out in front of the animal and *Y* points vertically upwards. Positive azimuth is an anti-clockwise rotation about the *Y*-axis. In this coordinate system, points within the binocular overlap have positive disparity: larger for nearer objects, and falling to zero for points at infinity. The dashed circles represent the spatial receptive fields of sensors which are tuned both to disparity, and to visual direction (azimuth and, in a 3D model, elevation). (*c*) Two-dimensional retinal images (i.e. showing both azimuth and elevation). The star is assumed to be at zero elevation in both eyes, and its azimuth in each eye is shown.

Figure 1*a* illustrates the basic geometry of binocular stereopsis, while figure 1*b* introduces a convenient remapping used throughout this paper, where the visual directions in each eye are represented as Cartesian coordinates. The point marked with the orange star projects to azimuth *α*_L_* in the left eye and *α*_R_* in the right. This is shown in the two-dimensional retinal images, figure 1*c*; cross-sections through these retinal images are also shown in figure 1*b*, rotated to align with the axes there. On the main axes of figure 1*b*, the star is drawn at a position corresponding to *α*_L_* on the red *α*_L_ axis and *α*_R_* on the green *α*_R_ axis. Its visual direction can be defined as the average of the two, *α*_H_* = (*α*_R_* + *α*_L_*)/2. As the star is close to the observer, the angle *α*_R_* is substantially more positive than *α*_L_*, so it has a large disparity δ* = (*α*_R_* − *α*_L_*). The horizontal blue line in figure 1*b* marks all locations with this disparity.

Figure 1 is appropriate for an animal whose eyes are fixed in place on the head, like an insect, where vergence cannot contribute to depth perception [45]. The area shaded purple represents the binocular overlap. Each location in this region of space, e.g. the one marked by the orange star, corresponds to a different pair of visual directions in each eye: (*α*_R_,*α*_L_). Thus, if we can identify pairs of retinal locations which are viewing the same object, we can derive that object's location in the 3D space surrounding the animal.

The challenge, of course, is identifying pairs of retinal locations which are viewing the same object. This *correspondence problem* is a major area of research in both computer vision and visual neuroscience [33,46–49], to the extent that the distinguished computer scientist Takeo Kanade is said to have identified the three most important problems in computer vision as ‘correspondence, correspondence, correspondence!’ [50]. Stereo correspondence algorithms aim to, for a given point in the left eye, either find the matching point in the right or classify the left-eye point as unmatched, e.g. due to monocular occlusions. This produces a disparity map across the scene.

### (a) Local disparity sensors and different types of local correspondence

The first step of essentially all stereo correspondence algorithms is to establish some local cost function, which measures how well the left and right images ***I*** match within a small window ***W*** around the points under consideration. The small dashed circles in figure 1*b* each represent one such window, each centred on a particular pair of locations (*α*_R_,*α*_L_) in right, left eyes. I will use the term ‘local disparity sensor’ to refer to a window plus some way of assessing the degree of correspondence between image-patches, i.e. how well the left and right image-patches match. That is, a local disparity sensor performs some function combining the left and right images and windows, so as to produce a scalar value for a given image-pair.

Bioinspired and machine stereo algorithms differ in how they assess correspondence. In elucidating these differences, it will be useful to categorize local disparity sensors as implementing ideal, strong, weak or no correspondence, depending on how sensitive their output is to the degree of match between left and right image-patches.

#### (i) Ideal local correspondence

I will define a disparity sensor doing *ideal* correspondence as one that produces a pure measure of how well the two image-patches match. All matching image-patches elicit the maximum response, unconfounded by other image properties such as contrast or whether the patch contains particular features. Non-matching image-patches elicit a lower response.

#### (ii) Strong local correspondence

A disparity sensor doing *strong* correspondence is sensitive to the degree of match, but this is confounded with other properties. Unlike an ideal disparity sensor, different perfectly matching images may not elicit the same response. But mismatching image-patches elicit a smaller response even if they are intrinsically equally attractive. So for example a sensor doing strong correspondence may give a stronger response when viewing a face in both eyes, than when viewing a house in both eyes. But even if it happens to respond equally well to a house in both eyes as to a face in both eyes, to qualify as doing strong correspondence, it must respond less well when presented with a house in one eye and a face in the other.

#### (iii) Weak local correspondence

Now consider a disparity sensor where this is not the case: if a binocular house and binocular face give the same response, then a dichoptic house/face will also give this same response. Such a disparity sensor clearly does not qualify as performing strong correspondence. However, it may still qualify as doing *weak* correspondence if it responds more *on average* to matching images, when one considers matching versus non-matching pairs drawn from the ensemble of all ecologically relevant images.

#### (iv) No local correspondence

A disparity sensor where there is no difference even on average between matching and non-matching images does not qualify as doing correspondence.

Having defined these terms, let us see how the different types of disparity sensor produce different types of correspondence. Let us represent both images and windows by vectors: ***I***_L,R_ for the images in the two eyes and ***W***_L,R_ the window functions. The machine stereo literature generally uses binary windows, i.e. the vector elements of ***W*** are 1 inside the window and 0 elsewhere, and element-wise (Hadamard) multiplication of the image with the window, which I denote ${\;W}^{\circ }I$. Common local cost functions are the sum of absolute differences, $-|W_{L}^{\circ }I_{L}-W_{R}^{\circ }I_{R}|$, sum of squared differences, $-{\left|W_{L}^{\circ }I_{L}-W_{R}^{\circ }I_{R}\right|}^{2}$ and normalized correlation, $\left(W_{L}^{\circ }I_{L}).(W_{R}^{\circ }I_{R})/|W_{L}^{\circ }I_{L}||W_{R}^{\circ }I_{R}\right|$ [51] (the minus signs in front of the first two metrics are just to ensure that all three metrics measure goodness of match, not badness). These can be viewed as the output of different types of local disparity sensor. The negative sum of absolute or squared differences are zero for all matching image-patches and less than 0 for non-matching, while normalized cross-correlation is 1 for all matching image-patches and less than 1 for non-matching. Thus, all three satisfy the criteria for ideal correspondence: all matching image-patches elicit the maximum response, unconfounded by contrast, etc., while non-matching image-patches elicit a lower response. Thus, machine stereo is based overwhelmingly on ideal local correspondence.

In current models of biological stereopsis, the disparity sensor is a binocular neuron and the window functions ***W***_L,R_ represent its left and right receptive field functions. These are not binary but are usually Gabor-like functions including both positive and negative values. The response of the neuron is assumed to be some function of the inner product of the image with the window function, W.I.

**Figure 2.**
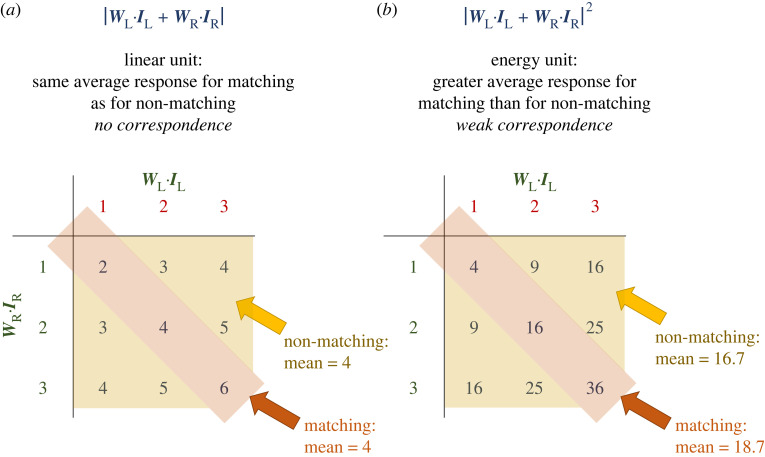
Toy example to show intuitively why a purely linear sensor (*a*) does not do correspondence, while a squaring output nonlinearity (*b*) converts it to doing weak correspondence. This is for a highly reduced stimulus ensemble where there are only three possible values of the monocular inner products: 1, 2, 3, which for simplicity we will assume to have equal probability. Neither of these units does ideal correspondence, because they respond more to non-matching images for which the inner products are 2 and 3 than to matching images for which the inner products are both 1. The linear unit is completely insensitive to correspondence. However, the energy unit shows weak correspondence, in that on average across all images, matching images evoke a stronger response than non-matching images. Note that a threshold can have a similar effect. For example, if one thresholded the linear unit such that responses less than 4 are set to 0, the mean response to non-matching images would be <{0,4,5} > = 3, while that to matching images would be <{0,4,6}> = 3.3. Units which respond differently to matching than to non-matching patterns show tuning to image disparity, since image-patches whose disparity equals that of the unit's receptive fields match, while image-patches with different disparity do not.

The simplest bioinspired models have considered sensors that do not implement correspondence at all, such as purely linear binocular mechanisms computing $\left(W_{L}.I_{L}\pm W_{R}.I_{R}\right)$ [52–54]. A sensor like this has a preferred disparity defined by the monocular receptive fields, and it is sensitive to the disparity of an isolated target. However, it does not show strong correspondence because in general, there are multiple images which elicit the same monocular responses, and the sensor responds equally to any pairs of these, whether or not they match. Furthermore, it does not even show weak correspondence because, as shown for a toy example in figure 2*a*, on average it responds the same for matching as for non-matching images. This means it cannot show disparity tuning in cyclopean stimuli like dynamic random-dot patterns, which effectively compute the mean response to many images with identical monocular statistics but different degrees of binocular match. Even if we make the monocular computation more general, $\left(\;f_{L}(I_{L})+f_{R}(I_{R})\right)$ where the functions *f*_L_ and *f*_R_ are may be nonlinear, the same problem applies.

However, adding an output nonlinearity after binocular combination can enable such a sensor to perform weak correspondence. The stereo energy model, introduced to describe neurons in cat visual cortex [55], achieves this. It is built from binocular simple cells that compute ${\left|W_{L}.I_{L}\pm W_{R}.I_{R}\right|}^{2}$. The squaring output nonlinearity makes the binocular simple cell sensitive to correspondence on average, producing disparity tuning with dynamic random-dot patterns. Figure 2*b* provides a simple example to give intuition about why this occurs; see appendix D of [56] for a formal proof that this model produces disparity tuning (and that the disparity tuning curve obtained with dynamic noise patterns is approximately proportional to the cross-correlation of the receptive field functions). Other output nonlinearities, for example thresholding, produce a similar response. However, the energy model simple cell still shows only weak correspondence, because the cell's response is unchanged by swapping one monocular image for a different one which produces the same monocular activation even though it does not match binocularly.

Combining several such energy-model simple cells produces an energy-model complex cell, which starts to move towards strong correspondence. Intuitively, this is because as more simple cells are added, it becomes progressively more challenging to swap in a different monocular image which produces the same activation in all units. The large response of V1 neurons to their preferred disparity [57–59] and their lack of response towards ‘anti-correlated’ stimuli with artificially low matches [60] has recently been modelled by a convolution model combining very many simple cells [61]. Repeated iterations of this process may also explain the properties of neurons in higher areas such as IT, where the response to anti-correlated stimuli is nearly abolished [62]. However, no biological neurons have been yet identified that implement ideal correspondence.

### (b) Perceiving depth via a population of disparity sensors

In machine algorithms, there is typically no fixed set of disparity sensors. The disparity between left and right windows is usually a continuous variable input to an ideal correspondence function, which is adjusted until the best match is found. In adaptive-window machine algorithms, the size of the matching windows is also varied in response to the scene structure, aiming to make the windows large enough to avoid ambiguity in, for example, densely textured surfaces, while keeping them small enough that the disparity is approximately constant over a window [63,64].

In biological models, it is usually assumed that local disparity sensors correspond to a population of disparity-tuned neurons, like that represented by dashed circles in figure 1*b*. For each visual direction, there are many sensors, each tuned to a different disparity. The available sensor disparities are thus discrete, depending on the population properties. Window (i.e. receptive field) sizes may vary with luminance/contrast adaptation state, but there does not seem to be adaptive-window variation depending on scene disparity. The estimated scene disparity at each direction is encoded by the preferred disparity of the most active sensor. As a consequence, the resolution of primate stereopsis is low, limited by the available receptive field sizes [65–68].

The vision scientist Walls [69] argued that binocular single vision is essential and intimately linked to stereopsis: any animal with binocularity must ‘see the object singly with two separate eyes [i.e.] they must have fusion of the two images of the object’ (requiring correspondence), and ‘have parallactic localization of the object in space’, since ‘[Animals] would gain absolutely nothing from binocularity if they saw the object diplopically’. I shall argue below that this assumption is unwarranted.

**Figure 3.**
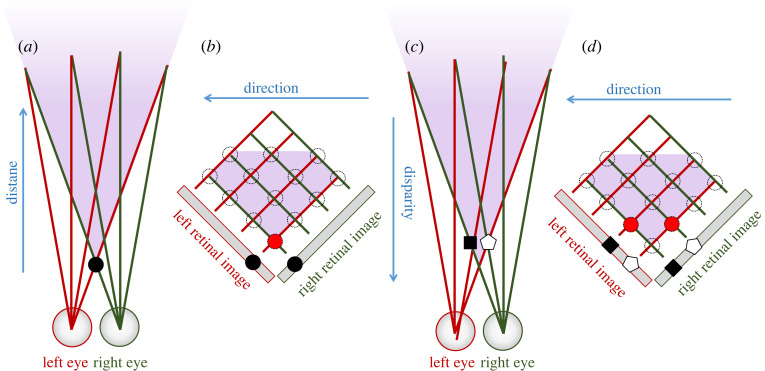
Local correspondence suffices for sufficiently unambiguous stimuli. This toy example considers a fixed-eye visual system with just four locations in each eye and 13 pairs of locations which correspond to locations in space, defined by the intersection of lines of sight from left and right eyes (in AC, the three pairs corresponding to infinite distance are not visible). The dotted circles in BD represent the 13 disparity sensors which compute the degree to which left and right eye images match at their location. Sensors which are experiencing high local correspondence are filled in red. Note that because in this example, the visual fields extend further medially than temporally, the binocular overlap region (shaded purple) is a truncated diamond in the Cartesian representation.

However, it is worth noting that even a population of biological disparity sensors, which as we have seen do not achieve ideal correspondence, still implements Walls' binocular single vision in sufficiently simple situations. For example, the object in figure 3*a* appears to the right of the midline in the left eye and to the left in the right eye. However, the disparity sensor it activates has its cyclopean location on the midline. Thus, the brain can successfully perceive a single object straight ahead, instead of two objects on either side of the midline.

Figure 3*c*,*d* shows a slightly more complex situation in which two objects are present, represented by a black square and a white pentagon. Let us assume that the particular disparity sensors under consideration have weak correspondence and are activated when both receptive fields see the black square or when both see the white pentagon, but not when one eye sees the black square and one eye sees the white pentagon. (As a concrete example, suppose that in both eyes ***W*.*I*** = −2 for the black square and +2 for the white pentagon, and that the disparity sensors compute ${\left|W_{L}.I_{L}+W_{R}.I_{R}\right|}^{2}$). In this case, as shown in figure 3*d*, only the two disparity sensors encoding the spatial locations of the two objects become active: both at the same depth, one on either side of the midline. Thus for this example also, the population successfully implements both binocular fusion and depth perception. However, this depends critically on successful local correspondence—that is, that only the two sensors corresponding to the location of the physical object become active. For biological sensors, this is certainly not guaranteed.

### (c) Local versus global correspondence

**Figure 4.**
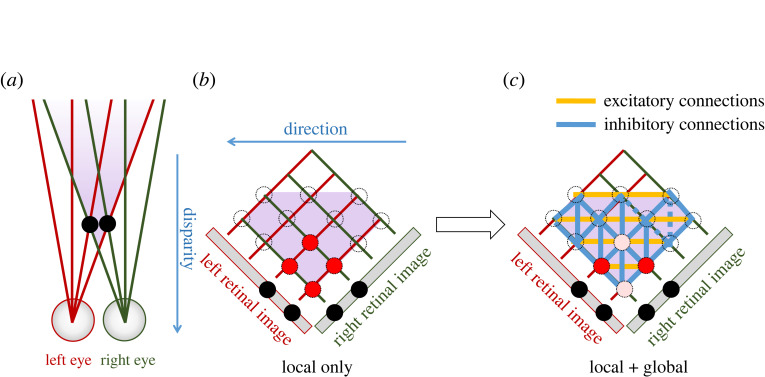
Global correspondence. (*a*) Top-down view of a scene containing two objects. (*b*) Cartesian array of disparity sensors; those shaded red would be activated by feed-forward activity from the two eyes, signalling a local match between left and right images within the sensor's window. (*c*) After adding recurrent connections, as shown ((*c*) could also be regarded as the activity in a higher brain area receiving input from the layer shown in (*b*), with the weights indicated). Stereo algorithms often implement mutual excitation between disparity sensors tuned to similar disparities at different cyclopean locations (shown here by gold horizontal lines). They may also postulate inhibitory connections between disparity sensors corresponding to the same location in one eye (oblique blue lines, as in [72]), and/or between disparity sensors corresponding to the same visual direction (vertical blue lines, as in [71]).

If the disparity sensors are not able to distinguish between the two objects, four disparity sensors would become active instead of two, as shown in figure 4*a*,*b*, and so four objects would presumably be perceived—a form of double vision which Walls argued [69] would be devastating to the organism. To some extent, this situation can be avoided by more sophisticated local correspondence. However, in complex real-life scenes, there are often many small patches which are close to identical on the retina though created by different objects. In this situation, even if the local correspondence is ideal, many disparity sensors would become active, as in figure 4*a*,*b*. This is a real problem for both machine and biological stereopsis.

To successfully achieve correspondence, fusion and binocular single vision, we can impose various constraints which take account of the wider pattern of matches across several different sensors. For example, Marr & Poggio [72] postulated excitatory connections between sensors tuned to the same disparity (horizontal yellow lines in figure 4*b*,*c*), and inhibitory connections between sensors with receptive fields in the same location in one eye (oblique blue lines). This builds in a preference for *smoothness* (gradual change in disparity with azimuth) and *uniqueness* (each retinal location is assigned only one disparity). The influential early stereo algorithm PMF [70] used a disparity gradient limit to achieve a similar result, while a recent stereo vision system using event-based sensing and spike-based neuromorphic hardware [71] has each disparity sensor receiving feed-forward excitation from a ‘coincidence neuron’ tuned to the sensor disparity, but feed-forward inhibitory input from all other coincidence neurons tuned to the same cyclopean position (vertical blue lines in figure 4*b*,*c*). In machine stereo algorithms, this is usually achieved in an energy-minimization framework [33,51]. I will refer to all such interactions as implementing ‘global correspondence’, because they occur over a wider area than the window used by the local disparity sensor, although in biological systems ‘long-range’ might be more accurate than ‘global’, since the interactions may not extend over the whole visual field. These ‘global’ interactions ensure that the initial activation of figure 4*b* results in the final activity of figure 4*c*: the pair of local disparity sensors which reciprocally excite each other remain active, while the pair they inhibit are suppressed.

### (d) Complications of mobile eyes

**Figure 5.**
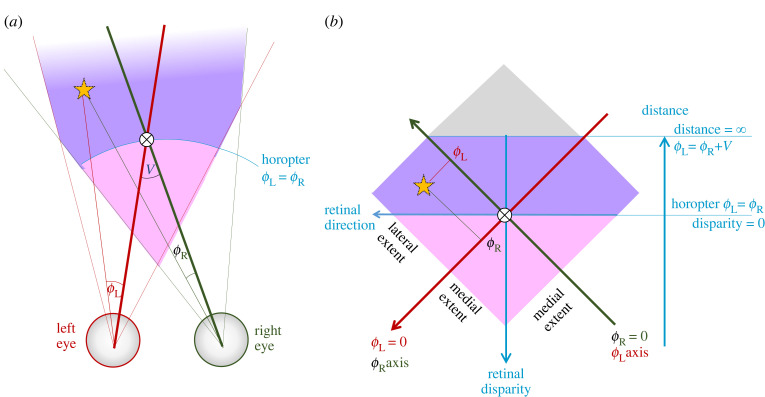
Binocular geometry modified for an animal, such as a human, with mobile eyes. *ϕ*_L_, *ϕ*_R_ represent retinal location relative to the fovea. The symbol ⊗ represents the fixation point. *V* is the vergence angle. The shaded region again indicates the binocular overlap, but now points nearer and further than the geometrical horopter are shaded in different colours. Note that to keep the sign convention the same as in figure 1, positive retinal disparities are defined to be nearer than the fixation point and negative those further away. This is opposite to many definitions in the literature.

So far, this discussion of biological stereopsis has not addressed a major complication affecting mobile-eyed animals like ourselves: the binocular overlap region moves in space. To reflect this, we need to modify figure 1 as shown in figure 5, replacing headcentric azimuth *α* with retinotopic azimuth *ϕ*, measured relative to the fovea. The binocular overlap region is now shaded in two different colours, with the pinker shade representing positive retinal disparities (closer than the geometrical horopter) and the bluer shade representing negative. With this modification, we can now consider retinotopic disparity sensors in exactly the same way as we have so far considered headcentric disparity sensors. The mapping from retinal disparity to location in space is now more complex, requiring a knowledge of eye posture as well as retinal location, but this will not affect our discussion of correspondence.

### (e) Primate stereopsis

Perhaps as a consequence of our mobile eyes, humans have at least two forms of stereoscopic vision, variously described as contour/coarse/transient/qualitative/headcentric, versus cyclopean/fine/sustained/quantitative/retinotopic [73–80]. The coarse, contour form of stereopsis operates over a wide range of disparities, but requires obviously identifiable monocular stimuli, such as a vertical bar in each eye. It may exist predominantly to enable vergence eye movements, ensuring both eyes fixate on the same object and thus enabling our fine, cyclopean stereopsis.

This is required because cyclopean stereopsis works only for a small range of disparities, of order a few degrees, around the central 20° or so of the visual field. This limit presumably reflects the computational cost of such smart stereopsis, which makes it unfeasible to implement it across the entire binocular overlap region. Within its limited range, cyclopean stereopsis operates in arbitrary images, including those in which there are no recognizable monocular images. Monocular occlusions are readily recognized and incorporated into the depth percept. We are poor at reconstructing metric depth from purely stereoscopic information [39,81], apparently because our visual system does not extract a precise estimate of eye posture, as would be needed to recover metric depth [82–84]. We do however seem to acquire a dense disparity map across much of the central visual field, with extremely high resolution in depth, although relatively low resolution in visual direction [65–68].

**Figure 6.**
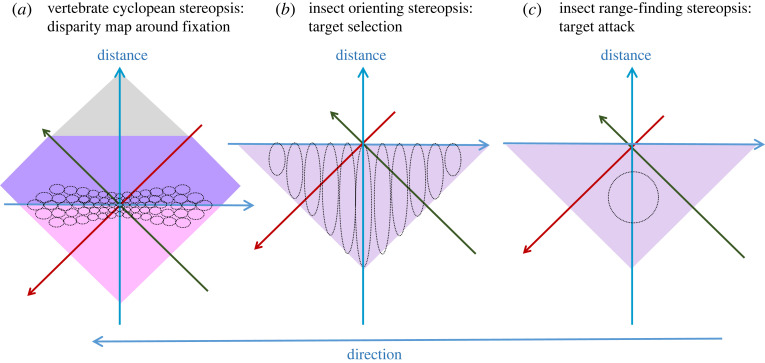
Sketch of disparity sensors for three different biological situations: (*a*) for a mobile-eyed animal aiming to produce a disparity map around fixation, based on monkey neurophysiology; (*b*) for a fixed-eyed animal aiming to take distance into account when orienting towards stimuli; (*c*) for a fixed-eyed animal aiming to detect when a large, isolated object is in range. None of the drawings are to scale.

Thus for a more accurate picture of primate cyclopean stereopsis, we should replace the population of disparity sensors in figure 1*b* with something closer to that shown in figure 6*a*. The disparity sensor windows are not uniform, as sketched in figure 1*b*, but are smaller and more numerous closer to fixation and become larger and sparser further away, making our depth perception less precise there [46,85–87]. The windows are also now drawn as ellipses, to indicate that the sensors are more narrowly tuned to disparity than to direction [55,88].

### (f) Additional processing required for correspondence

**Figure 7.**
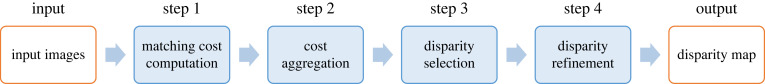
Four processing steps widely used in machine stereo algorithms. Redrawn from fig. 2 of [51]. Step 1, the matching cost computation, is typically local whereas steps 2–4 incorporate global mechanisms. (Online version in colour.)

**Figure 8.**
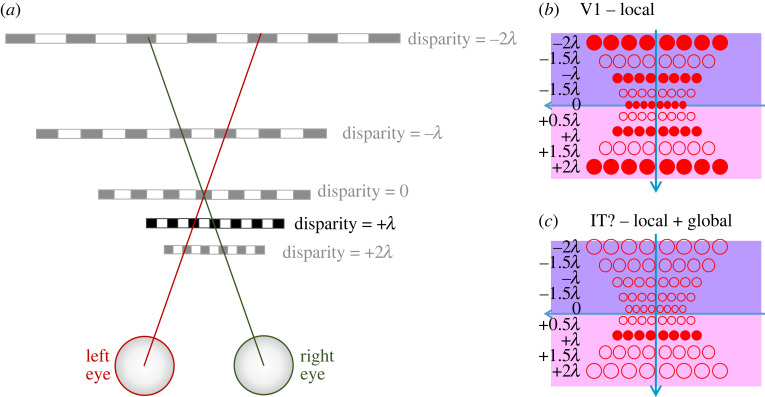
Ambiguous matching of a repeating stimulus. (*a*) The stimulus is a grating, with spatial period *λ* (measured as an angle, e.g. in arcmin). Its edges specify that it has a disparity of +1*λ*. For example, the left edge is at *α*_L_ = +2*λ* in the left eye and *α*_R_ = +3*λ* in the right. However, within a period of the fovea, this stimulus is locally indistinguishable from the other, fainter stimuli at disparities differing by multiples of *λ*. (*b*) Representation of disparity sensors in V1 (axes as in figure 5*b*), showing that neurons tuned to disparities of *Nλ* are all activated by this stimulus. (*c*) Putative higher brain area, perhaps IT, where activity corresponds to perception. Here, long-range interactions across the visual field have propagated the disparity defined by the edges across the ambiguous region. Perceptual resolution reflects the relatively coarse scale of the neurons tuned to disparity +*λ* in this neural-correlate area, rather than the fine scale of neurons tuned to zero disparity which are active in V1. In this sketch, for simplicity, I have shown the disparity sensor windows as circles rather than depicting their narrower tuning for disparity as in figure 6. (Online version in colour.)

For accurate stereo correspondence as in human cyclopean stereopsis, the initial combination of left and right images via local disparity sensors is just the beginning. We have already discussed the need for global mechanisms (figure 4*c*). Figure 7 shows the four main processing steps widely recognized in current machine stereo algorithms [33,51]. Similarly in primate stereopsis, an initial encoding of disparity in primary visual cortex (V1) undergoes extensive subsequent processing in further brain areas [46,48]. Our perception of depth is believed to reflect activity at the end of this process, perhaps in inferotemporal cortex [62]. The processing seems to reduce the responses to false matches (image-patches which are superficially similar but do not in fact correspond to the same point in space), contains specialized mechanisms for depth discontinuities, slant and curvature, and presumably also identifies monocular occlusions [89–94]. Similarly it seems clear that something like the global interactions discussed above do occur, but not exclusively within V1 itself [95]. For example, repeating patterns like sinusoidal gratings offer exactly the same local matches at disparities which are integer multiples of their spatial period *λ* (figure 8). The response of V1 neurons depends only on the local stimulus within the receptive field [96]. The +1*λ* stimulus shown in figure 8*a* thus activates V1 neurons tuned not only to +1*λ*, but to other multiples of *λ*, representing ‘ghost matches’ which are locally indistinguishable from real objects (figure 8*b*). However, humans (and monkeys, [97]) perceive this stimulus at the unique disparity indicated by its edges, +1*λ*, presumably reflecting activity in a downstream area where the purely local activity of V1 has been modified by more global processing (figure 8*c*). Intriguingly, the precision of depth judgements for such gratings reflects the usual low sensitivity associated with the relatively large disparity +1*λ*, rather than the high sensitivity found for judgements at the horopter [98], suggesting that the visual system is unable to exploit the activity of the high resolution, zero disparity local sensors active in V1.

### (g) Summary

In summary, then, despite important differences, primate cyclopean stereoscopic vision contains many conceptual similarities to an engineered dense stereo computer algorithm. Most importantly, at Marr's highest ‘computational’ level [99], both aim to solve the correspondence problem so as to achieve a map of disparity across a wide area of the visual field. At the algorithmic level, in both cases this begins with a computation of the match between local patches of the left and right images, which is subsequently refined to take into more global information from across the image, apply constraints, identify occlusions, propagate disparity across ambiguous regions and so on. This is a challenging and computationally costly process. In computers, stereoscopic vision is famously computationally costly, and much current effort is directed to ways of achieving it in real-time on constrained resources [100]. In primate brains, stereoscopic vision involves a complex network of many different brain areas [46,48]. This complex and sophisticated form of stereopsis has dominated research in the computing, psychology and physiological literatures.

## 3. Stereopsis without correspondence

I now consider what can be achieved by much simpler forms of stereopsis. Both these are inspired by praying mantis, the only insect to date in which stereopsis has been proven, notably in a beautiful series of papers by Rossel [6,28,101–103]. The praying mantis is a visual ambush predator, capturing prey with a ballistic strike of its spiky forearms. It uses stereopsis to detect when a prey item is in range. Before attacking, mantids make head saccades to fixate targets; in the words of Michael Land, ‘it is this primate-like behaviour that makes them seem so sinister.’ [104] These head saccades also show sensitivity to stereoscopic distance [103].

Intriguingly, the different properties of stereo-guided strikes and stereo-guided saccades suggest that these are controlled by distinct neural populations [103]. Head saccades can be triggered over a wide range of distances and eccentricities. Monocular and binocular targets attract saccades at the same rate, which could be taken as implying that binocular information is not used. However, if binocular targets are near enough to have different angles in the two eyes, the head saccades towards the average position—apparently fusing the locations [6]. Furthermore if there are two targets, equal in angular size and attractiveness but at different distances, mantids are more likely to saccade towards the nearer one [102]. If targets are artificially presented with a vertical disparity, mantids saccade towards the average vertical position, even if the vertical disparity is large [102].

This behaviour is quite different from the stereoscopically guided prey capture. There, strikes are extremely unlikely to be made towards monocular stimuli [105,106]. Strikes are not triggered towards stimuli with more than around 15° of vertical disparity [102]. Strikes are triggered mainly for a small range of distances and eccentricities: around 2–7 cm distance and within 20° of the midline [28].

As Rossel noted, these different behaviours suggest that mantids, like humans, possess at least two distinct forms of stereopsis [103]. To stretch the analogy further, in both species, one stereo system seems to be suitable for getting the eyes into the right position (turning to fixate the target, in the case of mantis saccades) and the other for subsequent depth perception (releasing the strike).

In the next two sections, I will sketch out possible designs for both systems. First, for the form of stereopsis guiding saccades, I will use a population of binocular neurons implementing $\left(\;f_{L}(I)+f_{L}(J)\right)$. Each neuron is tuned to a particular visual direction (azimuth and elevation, since this is a 3D model), and to a particular disparity. However, since binocular combination is linear, these neurons do not implement even weak correspondence, and since there is only one neuron for each visual direction, they cannot encode a map of disparity across the visual field, as in figure 1*b*. Nevertheless, I will show that they can produce the behavioural fusion and bias towards stereoscopically nearer objects shown by mantids. Second, for triggering strikes, I will use a single binocular neuron which does implement weak local correspondence. Despite being the only binocular neuron in the system, it can replicate effects traditionally explained by global correspondence. Although these systems were inspired by praying mantis stereopsis, they are not intended to offer a compete account of mantis stereopsis, but rather to demonstrate how apparently stupid algorithms can suffice for useful stereoscopic behaviour.

### (a) Stereoscopically guided head saccades

We first consider a stereoscopic system suitable for guiding head saccades, ensuring that, in a situation where multiple suitable targets are visible, mantids are more likely to fixate the one that is nearest.

#### (i) A simple two-layer network

The basic idea behind this model was described in a recent paper [8] and is sketched in figure 6*b*. I argued that linear combination of inputs from left and right eyes, followed by a winner-take-all step, could produce useful stereoscopic behaviour, such as turning towards the nearer of two potential prey items. The key to producing this behaviour was to have disparity-dependent synaptic weights from the monocular inputs onto the binocular neurons encoding direction. This toy model was two-dimensional (only zero elevation was considered); it received monocular input from eight locations in each eye, and chose one of five possible directions within the binocular zone. I showed (fig. 13 of [8]) that with hand-coded synaptic weights, the model made sensible decisions in four example situations.

**Figure 9.**
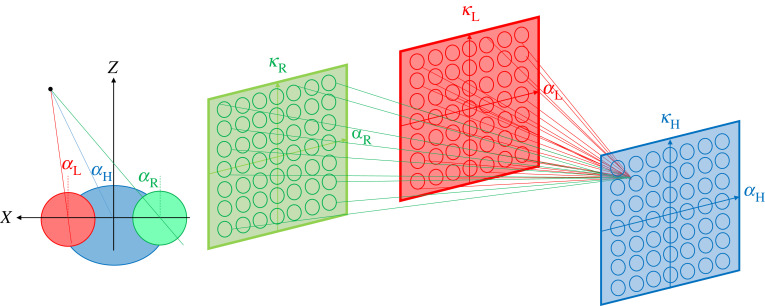
Stereoscopic network for controlling head saccades. In the monocular layers, each unit encodes a location in left (red) or right (green) retina. These layers then project onto a binocular output layer (blue), where each unit encodes a direction in which to turn the head. Lines show example connections onto one example output unit. (*α*,*κ*) are azimuth and elevation respectively; see Methods for details. (Online version in colour.)

I now instantiate this model more rigorously in three dimensions with synaptic weights learnt by training, and test it in a wider range of situations. The stereoscopic network consists of just two layers, the second fully connected to the first. In the second layer, each unit represents the direction (azimuth and elevation) of a head saccade. The saccade actually made corresponds to the direction encoded by the maximally active unit. In the first layer, each unit represents location (azimuth and elevation) in one of the two eyes. The activity in the first layer thus represents the result of earlier monocular processing which has identified potential prey items, for example the target-tracking algorithm described in [107]. This is not included in the model since our purpose is to evaluate the stereoscopic network. In this model, the output of the monocular stage is represented by bright target objects on a dark background, which form the input to the stereoscopic network (figure 9).

The output layer is fully connected to the units in the input layer. A head saccade is triggered in the direction encoded by the maximally active output unit. If the most active output unit is the one encoding zero, the head stays where it is.

**Figure 10.**
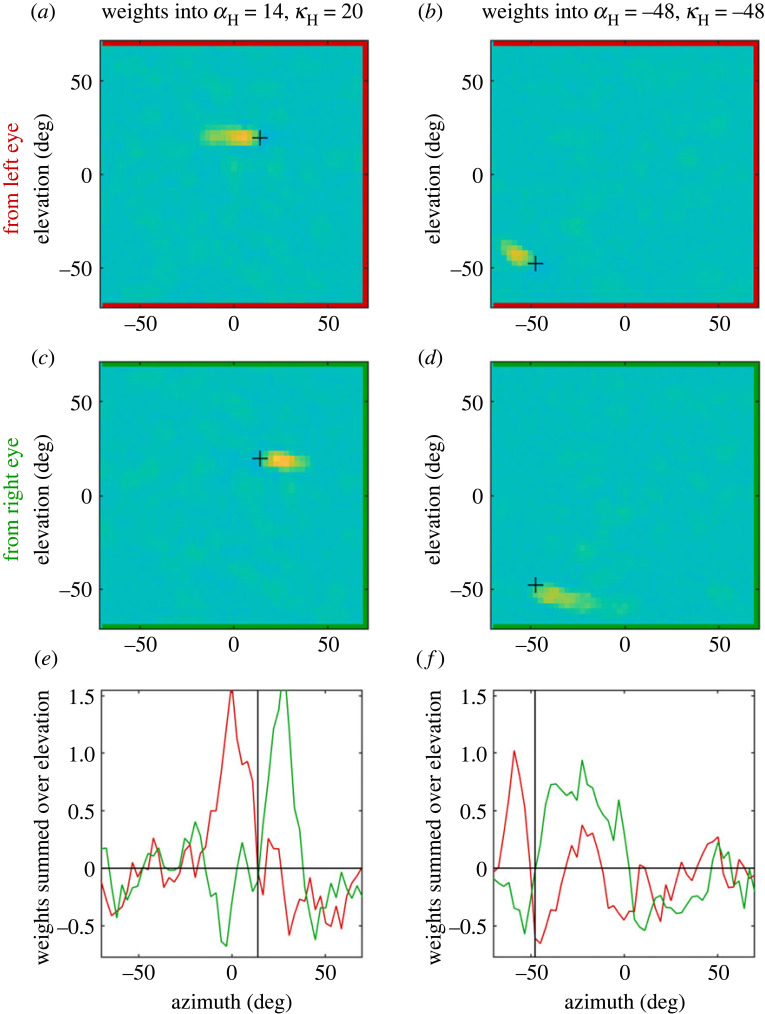
Network weights following training. The columns show weights into two example output units, representing the directions indicated at the top and marked with a cross +. AB, CD show weights from the input layer units representing left, right eyes, respectively. The colour axis is the same in all panels, and set such that zero is green, positive weights are yellow and negative are blue. EF show the total weights for each azimuth, summed over elevation, with weights from left (red) and right (green) eyes superimposed. This figure was generated by file Fig_ShowWeights.m. (Online version in colour.)

This simple two-layer network was trained on 100 000 example visual scenes, each containing four spherical objects. For each scene, the ‘correct’ head-rotation was defined to be the one which would fixate the nearest object. The diameter of the objects was scaled with distance, such that they all subtended 10° from the midpoint between the two eyes, forcing the network to use disparity rather than angular size as a distance cue. Figure 10*a–d* shows the weights following training, for two units in the output layer. The pseudocolour represents weights into the chosen unit from input units representing left (AB) or right (CD) retina. The weights are mainly zero, except in the vicinity of the direction encoded by the output unit (black cross), where there is a yellow smudge representing a region of excitatory weights. Notably, this excitatory region is offset in the two eyes. This is made clearer in figure 10*e*,*f*, which shows the total weight, summed over elevation, as a function of retinal azimuth; the black line represents the headcentric azimuth encoded by the output unit. In each case, there is strong excitation from the left-eye input units when *α*_L_ < *α*_H_, and weaker inhibition when *α*_L_ > *α*_H_; whereas there is strong excitation from the right-eye input units when *α*_R_ > *α*_H_, and weaker inhibition when *α*_R_ < *α*_H_. This matches the geometry shown in figure 1: for nearby objects, *α*_L_ < *α*_H_ < *α*_R_. The vertical structure seen in the second example, figure 10*b*,*d*,*f*, reflects another feature of the geometry: that eccentric nearby objects project to different elevation-latitudes on the two retinae, and thus the retinal disparity has a non-zero vertical component in our chosen coordinate system [108]. (This natural or epipolar *retinal* vertical disparity must not be confused with non-epipolar vertical disparity artificially applied to the *stimulus*, as in [102].)

Thus, the network has learnt a pattern of disparity-dependent synaptic weights similar to those hand-coded in the earlier toy model (cf. fig. 12 of [8]). The disparity-dependent weights seen in figure 10 mean that a nearby object, with *α*_L_ < *α*_H_ < *α*_H_, will generally excite the relevant output unit more strongly than a distant object with the same value of *α*_H_. A nearby object can produce strong excitation from both retinae, whereas a distant object of the same size will produce weaker excitation and some inhibition as well. We now examine how well this enables the network to perform in practice.

#### (ii) The network has learnt binocular fusion and a preference for nearer objects

**Figure 11.**
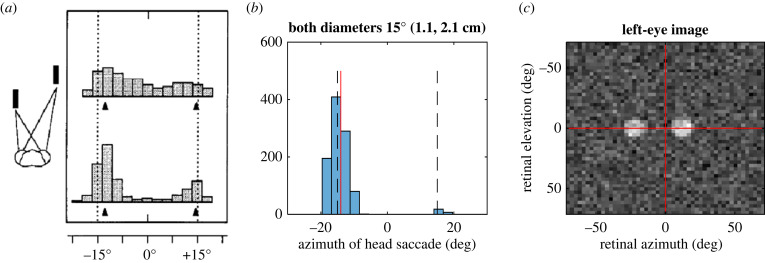
(*a*) This is a reproduction of figure 2*c* from [103], showing the distribution of mantis head angles after the first (top) and second (bottom) saccades. The targets are at ±15° azimuth (dashed lines), and equal elevation; the left target is 4 cm from the animal and right at 14 cm; both have the same angular size (4° × 20°) and same angular elevation. (*b*) Simulation results, in which the model views two spherical objects, at ±15° azimuth (dashed lines) and equal elevation; the left target is at 4 cm from the animal and the right at 8 cm; both have the same angular size (15° corresponding to physical diameters of 1.1 cm and 2.1 cm, respectively). The histogram shows distribution of (the first) head saccade for 1000 presentations of the (noisy) stimulus. (*c*) An example noisy monocular input image. (*b*,*c*) Produced with Matlab file AssessPerformance.m. (Online version in colour.)

Figure 11*b* shows results of a simulated experiment designed to mimic work by Rossel [103] which showed that mantids preferentially turn to fixate the nearer of two objects with identical eccentricity and angular size (figure 11*a*). Even though the objects are closer together in the simulation (4 and 8 cm from the head, rather than 4 and 14 cm), the model shows much stronger preference for the nearer object than the mantis, with virtually all saccades directed towards the nearer object. Note that the model also shows the same behavioural binocular fusion as the mantis. The centre of the nearer object projects to *α*_L_ = −22° and *α*_R_ = −8°, yet head saccades are made to *α*_H_ = −15° ± 1.6° (mean ± s.d.), i.e. towards the cyclopean location.

**Figure 12.**
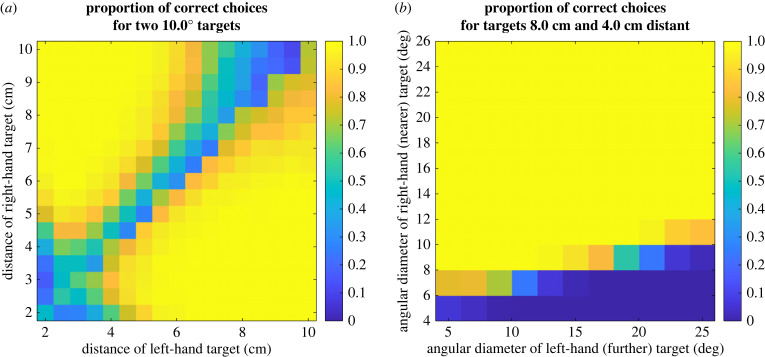
Model's preference to fixate near targets. Colour represents the proportion of trials on which the model made a saccade towards the nearer of two targets. The two targets were to left and right of the animal, at azimuth ±15°. (*a*) Targets varied in distance as shown on the axes, but their physical size was adjusted so that both targets always subtended the same angle (10°) on the retina. (*b*) The distances were fixed at 8 cm, 4 cm for left, right targets, respectively. These plots were generated by file AssessPerformance.m. (Online version in colour.)

Figure 12 quantifies performance across a range of such experiments, with targets again at ±15° azimuth but differing in size and distance. In figure 12*a*, the targets always both subtend 10°, but differ in distance. A ‘correct’ choice is defined as a saccade in the azimuthal direction of the nearer object (so, leftwards for points above the identity line, where the left target is closer). The model chooses the correct locations almost always when the target distances differ by more than 20% or so. Due to the particular pattern of weights learnt, this model displays a slight bias to choose the righthand target when the targets are both very close.

In figure 11*b*, the targets are fixed at 8 and 4 cm distance, but the plot shows results for varying diameters and thus angular sizes. When the nearer target subtends less than 6°, it is ignored and the larger, further target is always—wrongly—chosen (blue strip across bottom of panel). This also happens when the nearer target is much smaller on the retina: e.g. if the nearer target is 10° and the further target is 25°, the larger, further target is always wrongly chosen. However, the large expanse of yellow shows that the system is fairly robust to such size-based errors: unless the size difference is extreme, the system consistently picks the nearer target even if this appears smaller on the retina.

#### (iii) Model versus mantis

This model was inspired by praying mantis head saccades, but does not fully account for observations. For example, I noted above that when shown a target with non-epipolar vertical disparity, mantids saccade to the average of the vertical monocular locations. The present network usually, instead, chooses one of the two locations. This could suggest that the saccade direction represents the vector average of activity in the output layer, not its peak; separate winner-take-all steps could occur to select a target in each eye individually, though this would still need to be sensitive to disparity (i.e. to the choice of targets in the other eye) in order to produce the observed stereoscopic sensitivity. Thus, the mantis network is likely more complex than this simple model.

The point of this model is to provide an existence proof that a simple two-layer stereoscopic network can produce useful behaviour. To emphasize the extreme simplicity, I made the units entirely linear, rather than building the second layer from ReLU (rectified linear units), for example. In this case, this does not change the network performance, since the identity of the most active output unit would be unchanged by setting units with negative activity to zero (and since the inputs to the network are excitatory). However, it emphasizes that the binocular units in the output layer are not implementing even weak correspondence. The only nonlinearity occurs at the decoding level, where the population is read out so as to produce a single behavioural output.

### (b) Stereoscopically guided striking behaviour

We now turn to the second form of mantis stereopsis: that which triggers a strike when an object is in the catch range.

#### (i) A single disparity sensor

**Figure 13.**
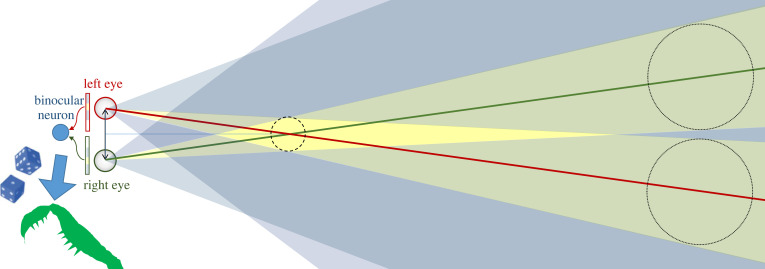
Properties of a binocular ‘disparity sensor’ with centre/surround monocular receptive fields. Top-down view of an animal, showing left and right eyes. On the left, we show one-dimensional cross-sections through the retinal receptive fields feeding into a binocular neuron, and on the right we project the receptive fields out into the space in front of the animal. Red, green lines mark the centre of the monocular receptive fields in each eye. In each eye, yellow colour-codes a central excitatory region and blue an inhibitory surround region. The monocular receptive fields are thus tuned to stimuli of a given angular size. In space, yellow regions are those which project to the excitatory regions in both eyes, blue to inhibitory in both eyes, and green those which project to the excitatory region of one eye's receptive field and the inhibitory region of the other. The optimal stimulus is indicated by the smaller dashed circle: this object falls in the central excitatory region in both eyes without stimulating any inhibitory region. The two larger dotted circles mark positions where two large objects could cause a ‘ghost match’ by producing the same image in each eye as the optimal stimulus (as well as a second image due to the other object). However, because these objects produce excitatory stimulation in only one eye, while inhibiting the other, they do not activate the binocular neuron. The dice and arrow leading from the binocular neuron to an image of the mantis fore-arm indicates that activity in the binocular neuron controls the probability that the mantis strikes. (Online version in colour.)

We recently showed that essentially all aspects of mantis stereoscopic strikes could be accounted for by a model incorporating just a single disparity sensor [44], as sketched in figure 6*c*. The model incorporates early monocular processing with low-pass spatial filtering to represent the sparse ommatidial spacing, and high-pass temporal filtering followed by rectification. This creates sensitivity to temporal change regardless of sign, as appears to be the case for mantis stereo [34,35]. The key features relating to stereopsis are illustrated in figure 13. A binocular neuron or ‘disparity sensor’ has a receptive field in each eye, positioned somewhat nasally. The inputs to the binocular neuron from each eye are given by the inner product of the receptive field functions with the processed stimuli, after the early monocular filtering. The receptive field functions have an excitatory-centre/inhibitory-surround structure, as has been observed for mantis binocular visual neurons [109,110]. This makes them size-tuned: they respond best to stimuli which are large enough to just cover the central excitatory region, without extending into the inhibitory surround.

This means that the optimal stimulus is an object of the optimal angular size, straight ahead of the mantis at the distance corresponding to the disparity of the receptive fields (smaller dashed circle in figure 13, in the lozenge-shaped yellow region of space where points project to excitatory regions in both eyes). This stimulus fully stimulates the excitatory region of both eyes' receptive fields, without producing any inhibition. By applying an expansive output nonlinearity, such that doubling the monocular inputs more than doubles the output of the binocular neuron, and using a threshold to prevent any response to weak inputs, we can ensure that the neuron responds only to near-optimal stimuli. For example, we can set the threshold such that the neuron does not respond to a stimulus visible in only one eye.

**Figure 14.**
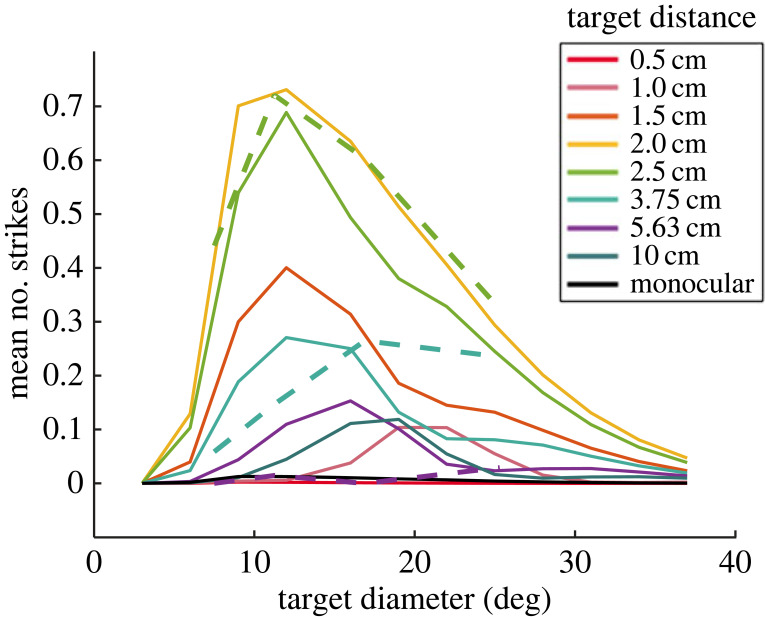
Size and disparity tuning of the model sketched in figure 13, reproduced from [44] but correcting an error in the empirical results as plotted in that publication. Solid lines show the mean number of strikes elicited from the model animal by a moving target at the distance indicated by the colour, and with the diameter indicated on the horizontal axis. Dashed lines show corresponding empirical results with praying mantises [111]. In the model, the mean number of strikes elicited by each trial is taken to be proportional to the time-averaged activity of the binocular neuron. (Online version in colour.)

Figure 14, reproduced from [44], shows how a model animal would respond to targets with a range of sizes and distances, if its probability of striking at any moment is proportional to the instantaneous activity of this simple binocular neuron. The three dashed lines compare data from real mantises. Remarkably, this very simple, single-neuron model is able to reproduce the qualitative features of real mantis behaviour. The most strikes are elicited for stimuli of approximately 10° diameter, approximately 2.5 cm from the animal. The number of strikes falls off very rapidly with changes in distance (halving or doubling the distance produces less than half the number of strikes), and less rapidly with changes in size (halving or doubling the size reduces the number but not by half). The model even reproduces the interaction between size and disparity: larger angular sizes are preferred when stimuli are more distant (the opposite direction from that which would produce a constant preference for physical size, in centimetres). In the model, this occurs because of false matches made between the leading and trailing edges of the moving target (see [44] for details).

The model also reproduces mantis behaviour for stimuli with vertical disparity. This non-epipolar disparity cannot occur in real stimuli. Mantids strike as normal for stimuli with a small amount of non-epipolar vertical disparity, but cease responding once the vertical disparity exceeds around 15° [102]. Notably, this limiting vertical disparity is independent of target diameter, i.e. it does not depend on the degree of overlap between a target in the left eye and a target in the right. The model reproduces this behaviour; the limiting vertical disparity is set by the size of the receptive field excitatory-centre, and is thus independent of the stimulus diameter.

#### (ii) Global correspondence and the ‘ghost match’ geometry

**Figure 15.**
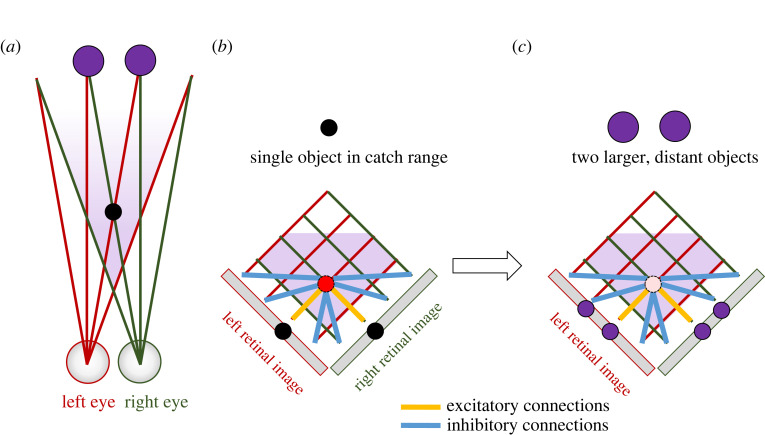
‘Ghost match’ geometry. Two identical distant objects (purple dots) create the same local retinal image as a single, smaller, nearer object (black dot), plus a second image in each eye. BC: Model containing a single disparity sensor. The excitatory and inhibitory connections onto this from the monocular images have a similar effect to the connections between pairs of disparity sensors in figure 4*c*, in that they prevent the sensor from being activated by the ghost match. (Online version in colour.)

This single-sensor model also displays surprisingly sophisticated behaviour when presented with more than one target. As shown in figures 13 and 15*a*, two identical large, distant objects can create the same local monocular retinal images as a single near object, although they also present a second stimulus in each eye.

We refer to this illusory nearby object as a ‘ghost match’ rather than a false match, since the retinal images are indeed perfect local matches [8]. Dragonfly larvae which hunt stereoscopically can be misled into striking at two distant objects, out of the catch range, as if they perceive a single near object corresponding to the ghost match [112]. Praying mantids too sometimes strike at such ghost matches, but the strike rate is around half what it is when for a single object with this disparity [44].

The single-sensor model reproduces this behaviour [44], thanks to its centre/surround receptive fields. As shown in figure 13, the distant objects necessarily extend into the inhibitory surround region in one of the eyes. Thus, they lie in regions of space shaded green, where points project into the excitatory region of one eye's receptive field, but the inhibitory region of the other. Whereas a real object at the catch range causes the binocular disparity sensor to receive excitation from both eyes and no inhibition, the ghost object necessarily produces both excitation and inhibition, reducing the probability of a strike from 70% for a real object to 20% for the ghost (table 4, [44]). Thus, this model produces behavioural results reminiscent of global correspondence, but the underlying mechanism is different. Whereas global correspondence involves excitatory and inhibitory connections between disparity sensors at different locations, cf. figure 4*c*, here there is only a single disparity sensor, and the excitatory and inhibitory connections are from monocular neurons onto the lone binocular neuron, as illustrated in figure 15*b*,*c*.

#### (iii) Model versus mantis

**Figure 16.**
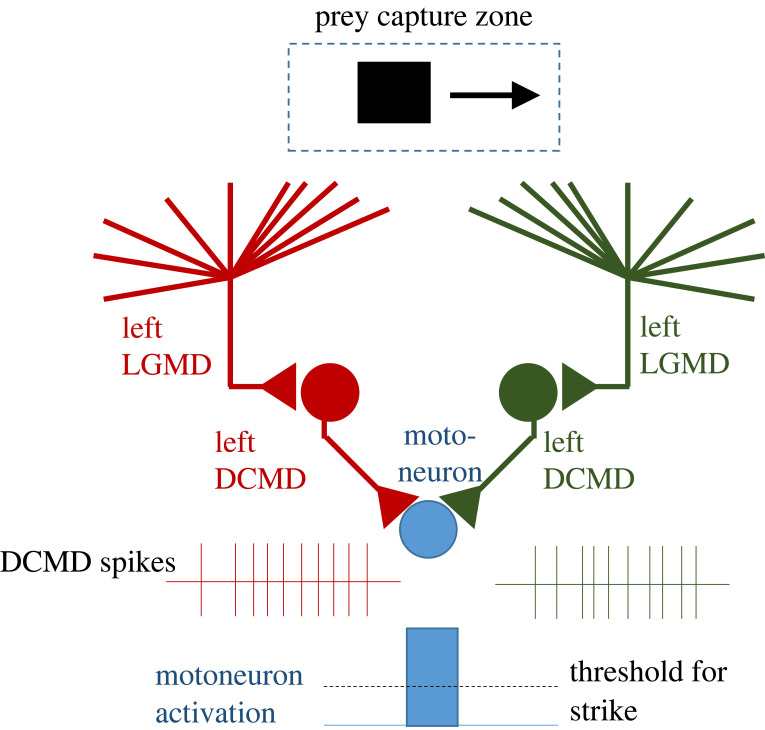
Schematic drawing showing model proposed by Kral & Prete [42] for mantis stereo-guided strikes. LGMD, lobula giant movement detector; DCMD, descending contralateral movement detector. Redrawn from fig. 3.15 of [42]. (Online version in colour.)

The model discussed here is very similar in concept to one sketched by Kral & Prete [42], sketched in figure 16, which also consists of a single disparity sensor. They postulated a pair of monocular lobula giant movement detectors (LGMD), which respond preferentially to preylike stimuli moving at a particular speed. These synapse, via a descending contralateral movement detector (DCMD) onto a motor neuron. Strikes are triggered when activity in the motor neuron exceeds a threshold. Kral and Prete argued that ‘a single pair of LGMD–DCMD complexes can explain how mantids identify, locate and strike at prey when it is centred in the visual field.’ They offer this model in opposition to the idea that ‘mantids use binocular disparity to judge the distance of an object’, or that mantids make ‘an explicit comparison of retinal images or calculation of retinal disparity’. I would argue that their model, like ours, does ‘use’ (is sensitive to) binocular disparity: the preferred disparity reflects the receptive field locations of left and right LGMDs. Furthermore, the threshold proposed for the motor neuron is a nonlinear operation implementing weak local stereo correspondence, which I would argue is a form of comparison of retinal images. However, we certainly agree that a single sensor cannot extract retinal disparity unconfounded by other stimulus dimensions. Concerning physiological correlates, the model of Kral and Prete postulates no binocular neurons in the mantis brain, with binocular information combined only in the thoracic motor ganglion. We now know that mantis brain contains multiple classes of disparity-sensitive binocular neurons, including some sending feedback to earlier visual areas [109,110]. Thus, the full circuitry actually subserving mantis stereopsis is in fact more complex than any of the models discussed in this paper.

**Figure 17.**
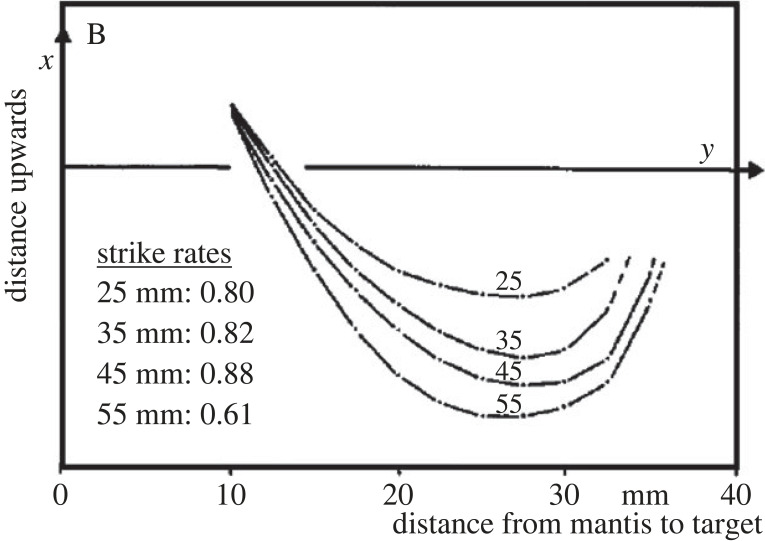
Mantids tailor strike trajectories according to stereoscopic disparity, for a given angular size. Reproduction of figure 3*b* from [101] showing data for a single mantis. The origin is the centre of the mantis head and the *y*-axis indicates the direction of the target. Curves show the path of the femur tip, each curve averaged over 30 strikes, for targets at stereoscopic distances of 25, 35, 45, 55 mm. The target was physically always at 55 mm with an angular diameter of 20°; nearer distances were simulated with prisms. Fifty-five millimetres is out of range and so no prey contact occurred during any of the strikes. I have superimposed the average strike rates for each distance, taken from of fig. 5 of [101]. These are the average over six animals, and it is not known whether these included the animal whose strike trajectories are plotted here.

As shown above, the single-sensor model gives a good account of current data on mantis striking behaviour, including the preference for size, vertical and horizontal disparity, and the lack of response to ghost matches. If so much is achieved by a single disparity sensor, why does the mantis brain have more than one? Most obviously, mantids do not strike solely at objects directly ahead of them. Having multiple disparity sensors, at different azimuths and elevations, could enable the animal to trigger strikes targeted towards the appropriate direction [113]. Secondly, a single disparity sensor necessarily confounds size and disparity, and indeed other stimulus dimensions like contrast or speed: for example in figure 14, the sensor is activated equally by a target at the preferred size but sub-optimal distance as by a target of sub-optimal size but preferred distance. Rossel [6,101] has provided compelling evidence that mantids do not confound size and disparity in this way. For example, when disparity indicates that a virtual object is very close, mantids may switch from attacking to defensive strikes [6]. Detailed measurements in one mantis showed that it systematically adjusted its strike trajectory depending on disparity, even when size is fixed ([101], figure 17). Importantly, the extent of the strike trajectory increases monotonically with distance, while the strike rate varies non-monotonically, which is not consistent with the idea that a single sensor is controlling both behaviours. This behaviour could be accounted for by multiple disparity sensors with different preferred disparities (and perhaps also sizes). The strike would be triggered by whichever sensor was most strongly activated and the strike trajectory would reflect the properties of the sensor triggering it.

Note, however, that even a population of many such disparity sensors could not encode a disparity map like that in figure 1*b*. The inhibitory surround zones, which produce the size tuning and inhibit the response to ghost matches, would also suppress responses to extended surfaces. Rather, this very simple form of stereopsis is suitable for judging the distance of large, isolated objects. Consistent with this, mantids strike only when prey is isolated in the temporal domain, e.g. via its motion or by change in luminance. In other words, mantis stereopsis likely works because early monocular temporal filtering extracts suitable targets from a complex scene, even though in the luminance domain prey may be perfectly camouflaged against the background [27,34,35]. There is no evidence that mantids perceive disparate objects in stimuli such as dynamic random-dot stereograms, where there is no monocular temporal difference between target and background, as humans and monkeys do.

## 4. Discussion

In this paper, I first discussed stereopsis as it is generally understood in the psychology and computing literature. Its aim is to achieve binocular single vision and a detailed map of disparity across a wide region of the visual field. This requires solving the stereo correspondence problem and is, therefore, extremely challenging. All neurally inspired attempts to model such an algorithm have proposed multiple disparity sensors for every visual direction, each tuned to a different disparity and sensitive to the degree of local match between image features at that disparity, as sketched in figure 6*a*. Physiological studies of vertebrate stereopsis have identified such neuronal populations in the visual cortex of monkeys, cats and mice, and in the visual wulst of owls [47,114,115]. These neurons show at least weak local correspondence, since they modulate their firing depending on the disparity of random-dot patterns. Thus, this general approach seems to be extremely widespread among the vertebrates.

I then considered two very different forms of stereopsis, both inspired by behavioural and neurophysiological findings in the praying mantis. First, I considered a system suitable for stereoscopically guided head saccades. This involved a population of binocular neurons, each tuned to a different location in the visual field but all with the same broad preference for near disparities, as sketched in figure 6*b*. In the model, I deliberately made these neurons insensitive to the local match between left and right images; their activity is simply the sum of their inputs, with no boost for matching inputs such as is provided, for example, by a squaring nonlinearity [55,59] (figure 2). These design decisions—no matching metric, only one disparity sensor at a given location, all sensors tuned to a similar disparity—seem extremely stupid by the standards of conventional stereo algorithms.

However, I showed that this simple system suffices for the kind of stereoscopic sensitivity seen in mantis head saccades. It demonstrates a marked preference to select the nearer of two competing targets, even when these subtend the same angular size (figure 11*b*). Performance is certainly not perfect, but it may be as good as is required. Real mantids only show a bias to fixate nearer objects; when the angular sizes are equal, they fixate the more distant object around one-third of the time ([103], figure 11*a*).

As noted, this system is not implementing even weak stereo correspondence, since it is completely insensitive to the degree of match. Yet the model also behaves as if it has binocular single vision: head saccades are made towards the midpoint of the left and right images. However, this ‘behavioural fusion’ does not mean that the model is actually implementing binocular fusion or single vision as humans experience it. In the model, a disparate object activates multiple binocular neurons, reflecting its different locations in left and right eyes. So, if the neural correlate of perception is activity in the binocular layer, then objects would be perceived in multiple directions, and yet useful behaviour is still produced, contradicting Walls [69]. If on the other hand the neural correlate of perception is the output of the final winner-take-all step that selects only a single visual direction for a saccade, then the winning object is perceived singly—but only a single object is perceived at all. This is similar to an earlier suggestion by Rossel [6] that mantis stereopsis ‘allows only one object to be perceived at a time’. The crucial difference is that in this model, the object selection occurs at a binocular level. In Rossel's proposal, each optic lobe independently selects the most attractive object visible to the ipsilateral eye [6]. This monocular selection cannot account for the bias to select the stereoscopically closer of two otherwise identical targets (figure 12).

Next, I considered a system suitable for triggering predatory strikes when prey is in the catch range. Here, we could assume that the head-saccade system has already ensured that the prey is already roughly directly ahead of the animal. For triggering strikes, greater accuracy is required, since mantids rarely strike at monocular stimuli or at prey which is out of their catch range [6,28,105,106]. Here, we found that we did need a disparity sensor which achieves weak correspondence: our model fitted a strong expansive output nonlinearity (raising to the power of 5). As well as greatly enhancing the response to optimal stimuli, this also boosts the average response to matching stimuli, relative to non-matching stimuli with the same average monocular value. This is formally very similar to the binocular simple cell, the building-block of current models of primate stereopsis. However, monocular processing before binocular combination renders the mantis disparity sensor insensitive to properties of the stimulus which are fundamental to primate stereopsis, such as the direction of local motion or the sign of contrast (bright versus dark) [34,35]. This again seems very poor design from the point of view of primate stereopsis and conventional machine stereo.

That said, the proposed stereo-guided strike system does implement at least a weak form of stereo correspondence. It also seems fair to argue that it achieves a form of binocular single vision for isolated objects, if we assume that activation of a disparity sensor triggers a percept of a single object at the visual direction represented by the disparity sensor, i.e. on the midline in our example, rather than the individual monocular images to left and right of the midline (cf. Figure 15). However, this system can hardly be said to ‘solve’ the correspondence problem. Behaviour which might appear to indicate something more sophisticated, such as not striking at the ghost objects which mislead dragonfly larvae, can be accounted for simply by the inhibitory receptive-field surrounds of the single disparity sensor. Because this model seeks only to estimate the probability that an isolated, large (10–30°) object exists at a given distance, it does not need to work with multiple objects and so can largely bypass the stereo correspondence problem [6,41–43,103].

Although, as noted above, the two models presented here are certainly a simplication of mantis stereopsis, it, therefore, seems that praying mantids may not possess a crude, low-resolution version of vertebrate stereopsis—a version of figure 6*a* with fewer, larger disparity sensors, operating on temporally filtered inputs rather than image contrast. Rather, current evidence is consistent with something even more basic, as sketched in figure 6*b*,*c*. Table 1 makes an attempt to compare and contrast the two stereoscopic systems found in primates and insects. This should be regarded as a set of ‘current best guesses' intended to stimulate future work and discussion, rather than as a list of established facts.

**Table 1.** Comparison between primate and insect stereoscopic systems. Many of the answers are currently unclear, and in particular those regarding insect stereopsis represent my current best guess rather than a well-evidenced fact.

property	primate contour stereopsis	primate cyclopean stereopsis	insect stereopsis for orienting to targets	insect stereopsis for striking at targets
purpose	control vergence	acquire scene depth structure	control head saccades	trigger predatory strikes
operates on	image luminance contrast	image luminance contrast	image temporal change	image temporal change
effect of anticorrelation	inverts depth percept	destroys depth percept	no effect	no effect
weak stereo correspondence	probably	yes, in V1	maybe not	yes, in several classes of neurons
strong stereo correspondence	no	yes, in higher brain areas	no	probably not
binocular single vision and global correspondence	no	yes (within limits of fusion)	no	no
works with static images	yes	yes	no	no
breaks camouflage	no	yes	no	no

One claim sometimes made is that the forms of stereopsis proposed here are ‘not really stereopsis’. Clearly, this is a matter of definition. Some researchers have emphasized the qualia associated with human stereopsis (e.g. ‘a compelling perception of solidity or three-dimensionality, a clear sense of space between objects and a phenomenal sense of realism’, [116]), but it is unclear how to test for this in non-human species or in machines. I prefer to define stereopsis as ‘the ability to gain information about the 3D structure of visual scenes by comparing information collected separately and simultaneously from different lines of sight to the same region of space’ [30]. This definition in terms of information processing, rather than perceptual qualia, makes it more suitable for application beyond humans. The definition is designed to be broad enough to include such different situations as: a machine stereo algorithm producing a metric distance map [49]; human cyclopean stereopsis, which may produce fine-grained information about scene structure but recover distance only up to an affine transformation [39]; neural signals which trigger vergence but are never consciously perceived; da Vinci stereopsis based on occlusions [117]; the system governing mantis head saccades, which may not extract distance at all but only produce a bias towards stereoscopically closer objects [6]; or even monocular stereopsis in species whose eyes permit that, for example the mantis shrimp *Squilla* [45]. These very different applications all rely on the same underlying geometrical principle, and it is helpful to have a common word to describe them.

Regardless of how well the two systems considered in this paper turn out to describe mantis stereopsis, they demonstrate the importance of considering simpler forms of stereopsis [6,41–43]. In particular, they demonstrate that a network can map directly from retinal inputs to stereoscopically informed behaviour, without requiring an intermediate stage at which disparity is extracted, let alone a geometrical reconstruction of a 3D scene. This possibility has been ignored in the psychology literature, because humans have the perceptual experience of a disparity map across large regions of the visual field; for example, we perceive surfaces and depth boundaries in random-dot stereograms. However, it is possible that human vision may also exploit the simpler approaches discussed here, e.g. in the control of vergence eye movements, or in qualitative stereopsis which may reflect ‘the operation of a distinct neural mechanism designed to provide crude depth estimates for diplopic stimuli’ [118,119]. The correspondence-free approach should also be considered in applications where computing power is limited, e.g. autonomous aerial vehicles. Several studies have demonstrated the value of stereopsis in controlling drone flights, e.g. [120–124], but all have a stage where they explicitly compute disparity. Skipping this step may enable more efficient stereoscopically guided control of behaviour.

## 5. Methods

Simulation details for the model of stereoscopically guided striking behaviour are available in [44]. Simulation details for the model of stereoscopically guided head saccades follow.

### (a) Coordinate systems

We need to translate between different coordinate systems, including a world-centred coordinate system W, a head-centred coordinate system H and coordinate systems for left and right eyes, L and R. We will represent locations in space by column vectors, using superscripts to indicate the object whose location is being described and subscripts to show the coordinate system being used. For example, vector $P_{W}^{H}$ represents the position of the head in the world-centric coordinate system. In this paper, the head does not move position but remains at the origin, so $P_{W}^{H}=O$, but we will write the equations for the general case*.* The head's position is defined to be the location of the midpoint between the two eyes, which are a distance *I* apart. In the simulations, *I* = 1 cm.

The rotation matrix $M_{W}^{H}$ specifies the pose of the head relative to the world-centred axes. We define this using azimuth-longitude $\alpha_{W}^{H}$ and elevation-latitude $\kappa_{W}^{H}$ (Fick coordinates), where again the sub- and superscript indicate that these are the azimuth and elevation of the head relative to the world-centred axes. Thus we have

5.1 $$M_{W}^{H}=\left[\begin{array}{ccc} \cos\alpha_{W}^{H} & 0 & \sin\alpha_{W}^{H}\\ 0 & 1 & 0\\ -\sin\alpha_{W}^{H} & 0 & \cos\alpha_{W}^{H} \end{array}\right]\left[\begin{array}{ccc} 1 & 0 & 0\\ 0 & \cos\kappa_{W}^{H} & \sin\kappa_{W}^{H}\\ 0 & -\sin\kappa_{W}^{H} & \cos\kappa_{W}^{H} \end{array}\right]$$

Note that we apply the elevation first, then azimuth, which is what makes these Fick coordinates. The rotation matrix $M_{H}^{W}$ is the inverse of $M_{W}^{H}$, and describes the orientation of the world-centred coordinate frame as seen from the head-centred frame. Similarly, $P_{H}^{W}=-M_{H}^{W}P_{W}^{H}$ is the origin of the world-centred coordinate frame in head-centred coordinates.

The nodal points of the left, right eyes are at

$$P_{W}^{L,R}=P_{W}^{H}\pm 0.5IM_{W}^{H}X$$

where the + holds for the left eye and − for the right, *I* is the interocular distance, and $X={\left[1,0,0\right]}^{T}$, where T indicates transpose.

The gaze vector ***g*** defines where the head is pointing. In headcentric coordinates, this is the *Z*-axis:

$$g_{H}=Z={\left[0,0,1\right]}^{T}$$

and in world-centric coordinates this is rotated to reflect the head's azimuth and elevation:

$$g_{W}=M_{W}^{H}g_{H}$$

### (b) Turning the head to fixate an object

An object with world-centric coordinates $P_{W}^{o}=[X,Y,Z{]}^{T}$ has headcentric coordinates

$$P_{H}^{o}=M_{H}^{W}(P_{W}^{o}-P_{W}^{H})=M_{H}^{W}P_{W}^{o}+P_{H}^{W}$$

The head-centred elevation and azimuth of the object are $\alpha_{H}^{o}$ and $\kappa_{H}^{o}$, defined by

5.2 $$P_{H}^{o}=Z_{H}^{o}\left[\begin{array}{c} \cos\kappa_{H}^{o}\sin\alpha_{H}^{o}\\ \sin\kappa_{H}^{o}\\ \cos\kappa_{H}^{o}\cos\alpha_{H}^{o} \end{array}\right]=Z_{H}^{o}M_{H}^{o}Z$$

where $Z_{H}^{o}$ is the distance of the object from the head and where the rotation matrix $M_{H}^{o}$ is

$$M_{H}^{o}=\left[\begin{array}{ccc} \cos\alpha_{H}^{o} & 0 & \sin\alpha_{H}^{o}\\ 0 & 1 & 0\\ -\sin\alpha_{H}^{o} & 0 & \cos\alpha_{H}^{o} \end{array}\right]\left[\begin{array}{ccc} 1 & 0 & 0\\ 0 & \cos\kappa_{H}^{o} & \sin\kappa_{H}^{o}\\ 0 & -\sin\kappa_{H}^{o} & \cos\kappa_{H}^{o} \end{array}\right]$$

To fixate this object, the head must adopt a new posture H′, such that

5.3 $$M_{W}^{H^{\prime }}=M_{W}^{H}M_{H}^{o}$$

That is, the rotation matrix describing the new posture H′ is the previous matrix postmultiplied by a matrix describing the azimuth and elevation of the object as viewed from the original head posture. The head position remains the same, i.e. $P_{W}^{H^{\prime }}=P_{W}^{H}$.

With the head rotated into the coordinate frame H′, the object now lies on the gaze vector, directly in front of the head.

Equation (5.3) defines the new world-centric head azimuth and elevation, $\alpha_{W}^{H^{\prime }}$ and $\kappa_{W}^{H^{\prime }}$, in terms of the previous values $\alpha_{W}^{H}$, $\kappa_{W}^{H}$ and the azimuth and elevation of the object as originally seen in the previous head-pose, $\alpha_{H}^{o}$, $\kappa_{H}^{o}$.

This has a particularly simple form in the two-dimensional situation. If for example the head rotates azimuthally but its elevation remains zero, equation (5.3) reduces to $\alpha_{W}^{H^{\prime }}=\alpha_{W}^{H}+\alpha_{H}^{o}$. This makes sense since if an object is currently, say, 20° to the left $\left(\alpha_{H}^{o}=20^{\circ }\right)$ then to fixate it, the head needs to rotate 20° to the left. In this paper, we consider a fully 3D model in which the head can change both azimuth and elevation, so the simplification does not apply and we, therefore, use the general equation (5.3).

### (c) Retinal images

We assume that the eyes are fixed on the head. We assume that each point on the retina—each ommatidium, as it would be in the case of an insect eye—represents a particular visual direction relative to the nodal point of that eye. Again, we encode this direction using azimuth-longitude *α*_L,R_ and elevation-latitude *κ*_L,R_, where the subscript L,R encodes the eye. The visual axis of both eyes, i.e. the line corresponding to zero azimuth and zero elevation, are defined to be parallel to the head's gaze vector ***g***. This means that the eyes and head have the same rotation matrix: $M_{L}^{W}=M_{R}^{W}=M_{H}^{W}$. As before, an object with world-centric coordinates $P_{W}^{o}$ projects to retinal azimuth and elevation $\left(\alpha_{L}^{o},\kappa_{L}^{o}\right)$ in the left eye,

5.4 $$P_{L}^{o}=M_{L}^{W}(P_{W}^{o}-P_{W}^{L})=Z_{L}^{o}\left[\begin{array}{c} \cos\kappa_{L}^{o}\sin\alpha_{L}^{o}\\ \sin\kappa_{L}^{o}\\ \cos\kappa_{L}^{o}\cos\alpha_{L}^{o} \end{array}\right]$$

where $Z_{L}^{o}$ is the distance of the object from the left eye.

In the figures, we represent the two retinae as flat planes with horizontal and vertical axes representing azimuth-longitude and elevation-latitude, just as a Mercator projection represents the Earth on a wall-map. Equation (5.4) specifies how points in space project onto the retinae. In our model, the monocular input layers consist of 51 × 51 units for each retina, equally spaced in azimuth and elevation from −70° to +70° with a spacing of 2.8° between units.

**Figure 18.**
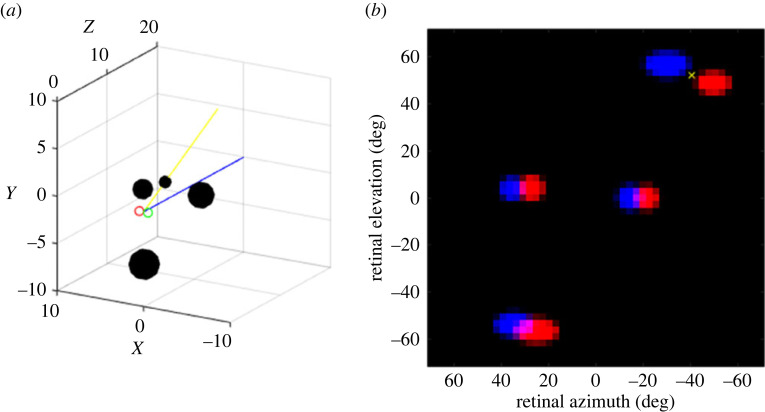
Example scene used for training. There are four objects, with headcentric azimuth-longitudes −40°, 31°, −17°, 29° and elevation-latitudes 52°, 4°, 0°, −54°, at distances of 3.7, 6.0, 7.8, 9.0 cm, respectively. Their diameter scales with their distance such that all subtend the same size, 10°, at the origin. (*a*) perspective view of the scene. *X*, *Y*, *Z* are the world-centric axes. The red and green disk mark the left and right eyes. The blue line is the gaze vector, here aligned with the *Z*-axis. The yellow line points from the origin to the closest object. (*b*) The left and right retinal images superimposed. Red, blue show the objects in left, right retinas. The yellow cross marks the headcentric azimuth and elevation of the nearest object. For correct behaviour, given this input, the most active unit in the output layer should be the one closest to this. In both panels, we are viewing from behind the head so the *X* and azimuth axes increase towards the left. This figure was generated by Make3DscenesConstAngSize_Fig.m. (Online version in colour.)

When we present simulated scenes to the network, we first project them onto the left and right retinae according to equation (5.4), and then trim/downsample to the spacing of the retinal units (cf. Figure 18*b*).

### (d) Network structure and training protocol

The retinal images form the input layer of our network, with 51 × 51 × 2 = 5202 units each representing a particular retinal location (*α*_L,R_,*κ*_L,R_). The output layer consists of the saccade-direction units, each representing a particular headcentric direction (*α*_H_,*κ*_H_), cf. Figure 9. We chose the same spacing as for the retina, i.e. 51 azimuths and 51 elevations, each equally spaced between ±70° with spacing 2.8°. The output units are linear units with weights and a bias. To compute a loss function, we applied a softmax function to the output layer and computed the cross-entropy loss for classification. The correct classification was defined to be the output unit representing the headcentric direction of the nearest visible object, as explained next.

We train the network by presenting simulated scenes consisting of a small number of spherical objects. These are projected onto the left and right retina according to equation (5.4). Figure 18 shows an example training scene (*a*) and the resultant retinal images (*b*). The closest object is at the top right, marked in yellow. The red and blue blobs indicating its projection in left, right images respectively are offset by a large amount, reflecting its proximity; distant objects project to the same locations in both eyes. Note that due to the Fick coordinates we have chosen, there is a vertical as well as a horizontal disparity [108]; this is just learnt and makes no difference to the behaviour of the model. The Fick (azimuth-longitude, elevation-latitude) coordinates also explain why the spheres at the top and bottom of the image appear elongated in the flattened representation of the retinal image, for exactly the same reason as Greenland appears elongated in the Mercator projection.

For training, we generated 100 000 noise-free scenes like the example in figure 18 (Matlab file Make3DscenesConstAngSize.m in the code repository). Each scene contained four spherical objects with azimuths and elevations generated independently from a normal distribution with mean 0° and s.d. 45°. Distances were generated independently from a uniform distribution over the range 1–11 cm. Diameters were scaled so that each sphere subtended 10° at the origin. Since the model retinas extended only to ±70° eccentricity, it was possible for some spheres to be out of view. For classification, the correct output unit was defined to be that representing the headcentric direction closest to that of the nearest visible object (strictly, the nearest object whose centre was visible). If no objects were visible, the correct unit was that representing (0,0), so that the head would stay in its current position.

To train the network, we used the function trainNetwork from the Matlab Deep Learning Toolbox with stochastic gradient descent with momentum, an initial learning rate of 0.05, a mini batch size of 128, and eight epochs with shuffling every epoch (Matlab file TrainBeast.m in the code repository). Examples of the trained weights are shown in figure 10. Bias was also trained but non-zero bias was learnt only for the output unit encoding no action (*α*_H_ = *η*_H_ = 0°), since this was the default action when no inputs were present. After training, bias was an order of magnitude larger for this unit than for any other unit.

## Data Availability

Matlab code is available at https://doi.org/10.25405/data.ncl.19487414.v1 [125].
